# Effect of deworming on Th2 immune response during HIV-helminths co-infection

**DOI:** 10.1186/s12967-015-0600-3

**Published:** 2015-07-18

**Authors:** Andargachew Mulu, Belay Anagaw, Aschalew Gelaw, Fuso Ota, Afework Kassu, Sisay Yifru

**Affiliations:** Department of Microbiology, College of Medicine and Health Sciences, University of Gondar, Gondar, Ethiopia; Institute of Virology, Leipzig University, Johannisallee 30, 04103 Leipzig, Germany; Department of Preventive Environment and Nutrition, Graduate School of Nutrition and Bioscience, Institute of Health Biosciences, The University of Tokushima, Tokushima, 770-8503 Japan; Department of Pediatrics and Child Health, College of Medicine and Health Sciences, University of Gondar, Gondar, Ethiopia

**Keywords:** Helminths, ART, Deworming, Th2, IgE, Ethiopia

## Abstract

**Background:**

Helminths infections have been suggested to worsen the outcome of HIV infection by polarizing the immune response towards Th2. The purpose of this study is to determine the activity of Th2 immune response by measuring total serum IgE level during symptomatic and asymptomatic HIV infection with and without helminths co-infection and to define the role of deworming and/or ART on kinetics of serum IgE.

**Methods:**

This prospective comparative study was conducted among symptomatic HIV-1 infected adults, treatment naïve asymptomatic HIV positive individuals and HIV negative apparently healthy controls with and without helminths co-infection. Detection and quantification of helminths and determination of serum IgE level, CD4^+^, and CD8^+^ T cell count were done at baseline and 12 weeks after ART and/or deworming.

**Results:**

HIV patients co-infected with helminths showed a high level of serum IgE compared to HIV patients without helminths co-infection (1,688 [IQR 721–2,473] versus 1,221 [IQR 618–2,289] IU/ml; P = 0.022). This difference was also markedly observed between symptomatic HIV infected patients after with and without helminths infection (1,690 [IQR 1,116–2,491] versus 1,252 [703–2,251] IU/ml; P = 0.047). A significant decline in serum IgE level was observed 12 weeks after deworming and ART of symptomatic HIV infected patients with (1,487 versus 992, P = 0.002) and without (1,233 versus 976 IU/ml, P = 0.093) helminths co-infection. However, there was no significant decrease in serum IgE level among asymptomatic HIV infected individuals (1,183 versus 1,097 IU/ml, P = 0.13) and apparently health controls (666 IU/ml versus 571, P = 0.09) without helminths co-infection 12 weeks after deworming.

**Conclusions:**

The significant decline of serum IgE level 12 weeks after deworming of both symptomatic and asymptomatic patients indicate a tendency to down-regulate the Th2 immune response and is additional supportive evidence that deworming positively impacts HIV/AIDS diseases progression. Thus, deworming should be integrated with ART program in helminths endemic areas of tropical countries.

## Background

An imbalance between a Th1 and Th2 cytokine profile with elevated serum immunoglobulin E (IgE) level was reported during acute HIV infection [1] indicating an abnormal T cell regulation of antibody synthesis by B cells. The presence of elevated IgE has been also correlated with disease progression and reported to signal the onset of opportunistic infections in patients with advanced HIV diseases and lowers CD4^+^ T cells [2, 3]. In sub-Sahara African countries where HIV-helminths co-infection is common, immune activation during HIV-helminths co-infection is complex [4–8]. The immune response of the host to helminths infection correlates with the production of interleukins (IL-4, 5, 9, 10, 13) and consequently the development of strong IgE response [2]. It is suggested that HIV- helminths co-infected individuals experience a marked shift from a Th1 response to a predominantly Th2 response [9–11]. This has been speculated to increase the risk of HIV transmission [10] and a cause for elevated plasma HIV RNA level and rapid diseases progression among sub Saharan Africa patients [11]. However, the administration of antiretroviral therapy (ART) [12, 13] and treatment of helminths infestations [10] showed a tendency to down-regulate the observed immune activation. We and others have observed an increased serum IgE level among diarrheic and tuberculosis patients [14–16] and a decline in serum IgE [14] and HIV RNA level [17–22] after treatment of helminths.

Despite the high rate of HIV-helminths co-infection and the benefit of deworming [9, 10, 14, 17–22], there is no routine screening for helminths infection and/or regular and/or mass deworming in most tropical countries including Ethiopia even for HIV/AIDS patients. There is little evidence regarding benefits of deworming in HIV-infected individuals receiving ART. Accordingly, we hypothesise that dewoming during initiation of ART will significantly reverse the elevated Th2 immune response. To explore this hypothesis, we present here preliminary data on the kinetics of IgE levels in vitro as an activity of Th2 immune response in symptomatic and asymptomatic HIV infection with or without helminths co-infection.

## Methods

### Patients

**Figure 1:**
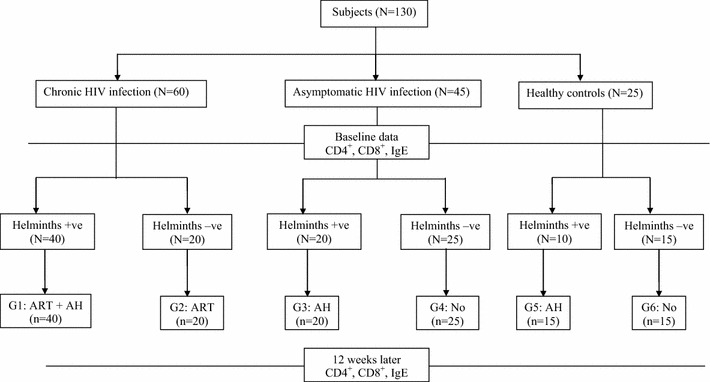
Cohort of HIV-1 infected individuals with helmitnhs and without helminths infection included in the study. Keys: *ART-* Antiretroviral drugs, *AH-* Antihelminthic drug.

This prospective comparative study was conducted at University Gondar Hospital, northwest Ethiopia among symptomatic HIV-1 infected adults at initiation of ART [with (N = 40) and without (N = 20) helminths co-infection], asymptomatic HIV positive individuals [with (N = 20) and without (N = 25) helminths co-infection] and HIV negative apparently healthy controls [with (N = 10) and without (N = 15) helminths co-infection]. The cohort profile is summarized in Figure 1. Patients enrolment was as follows: On one hand, consecutive clinically symptomatic patients with known HIV serostatus above 18 years of age, seeking treatment and willing to participate were evaluated with a standardized form at enrolment. On the other hand, detailed clinical history and complete physical examination including for any clinical signs as the WHO clinical staging of HIV infection were made for asymptomatic patients and enrolled when only if they were clinically asymptomatic and had no previous AIDS-defining conditions. Patients were excluded for the following reasons or conditions: pregnancy, treatment with single dose nevirapine for prevention of mother-to-child transmission of HIV or any other antiretroviral therapy (ART), known diabetes, hypertension, epilepsy, liver, cardiac and renal diseases, genital ulcer or active tuberculosis or atopic diseases (allergic asthma, allergic rhinitis or atopic dermatitis-according to self-reported and physician diagnosed allergy following the modified version of International Study of Asthma and Allergies module) at enrolment.

ART eligibility was based on the previous WHO recommendation with CD4^+^ T cell count of less than 200 cells/mm^3^ [23]. Accordingly, the 40 symptomatic HIV–helminths co-infected individuals started combined ART (D4T + 3TC + NVP; D4T + 3TC + EFV; ZDV + 3TC + NVP; or ZDV + 3TC + EFV) and also single dose 400 mg albendazole (Group-1, n = 40). Until the end of the study the level of adherence was optimal. The 20 symptomatic HIV infected individuals without helminths started ART (Group-2, n = 20). The asymptomatic HIV infected individuals were classified into 2 based on helminths infection: patients with (Group-3, n = 20) and without (Group-4, n = 25) helminths co-infection. HIV negative apparently healthy controls were also grouped into two: [with (n = 10), Group 5 and without (n = 15), Group-6 helminths co-infection]. Asymptomatic HIV infected individuals and apparently healthy blood donors with helminthic infection were dewormed with 400 mg albendazole. Moreover, 200 mg albendazole twice daily for 3 consecutive days was given for those found to have *Strongyloide stercoralis;* and 40 mg/kg praziquantel for those found to have *Schistosoma mansoni.* All subjects did not show any symptoms related to intestinal parasitic infection and atopic diseases (allergic asthma, allergic rhinitis or atopic dermatitis).

### Blood collection and analysis

At enrolment and 12 weeks after albendazole treatment, 5 ml venous blood was collected in vacutainer tubes containing EDTA. T-cells count was made using flow cytometer (FACSCount system; Becton–Dickinson, San Jose, CA, USA) following the manufacturer’s protocol. When clot is retracted serum was separated and stored at −40°C until used for investigations. The total serum IgE levels were quantified by the total IgE ELISA kit (IBL Immunobiological Laboratories, Hamburg, Germany) following the manufacturer’s instructions. Briefly, 10 ml serum samples or standard IgE were pipetted in duplicates into wells of microtiter plates precoated with monoclonal mouse antihuman IgE antibody together with per-oxidase conjugated antihuman IgE. After incubation for 30 min at room temperature the plates were rinsed with diluted wash buffer to remove unbound material. Then a substrate solution (tetra methyl benzidine) was pipetted and incubated for 15 min to induce development of colour. The reaction was terminated by the addition of stop solution and the resulting dye was measured in a spectrophotometer (Anthos Labtec Instruments, Salzburg, Austria) at a wave length of 450 nm against the substrate blank. The IgE concentration of the samples was read from a standard curve. Subjects found positive for intestinal protozoa (*Entamoeba histolytica* and *Giardia lamblia*) and Hookworm, *Taeniasaginata* and *Hymenolopis nana* were not included for IgE determination. This is because of different IgE responses observed in protozoal and helminths infections.

### Stool examinations

Stool samples collected at enrolment and 12 weeks after ART and/or anti-helminthic treatment were examined in 30–60 min using direct microscopy and formol-ether sedimentation techniques. Coarse quantification of eggs was made using the Kato-Katz method and a quantitative variable scoring (light infection/low worm burden, moderate infection/medium worm burden and heavy infection/massive worm burden) was created for each helminth following the standard procedure used by WHO [24].

### Statistical analyses

The data was analyzed using to SPSS version 17 statistical packages and GraphPad Prism 5 Software. Intestinal parasite densities were transformed to log_10_ for analysis and geometric mean was used. Data were summarized as medians and interquartile range (IQR). Non-parametric tests were performed to compare median serum IgE values of the different groups. The Mann–Whitney test and the Kruskall Wallis tests were used for comparisons between two groups and three or more groups, respectively. Spearman’s correlation was also used to check for correlations between parameters. P values was considered significant with <0.05.

### Ethical approval

The study protocol and design including the consent procedures were approved by Ethical Review Board of the University of Gondar, Ethiopia (Ref No: RPO/55/291/00). Written (from those who can read and write) or verbal (from those who can’t read and write) informed consent from all study subjects was also obtained. The written informed consent and the ethical clearance letters are documented. However, verbal consent was not recorded. Patient with HIV and/or helminthiasis was managed as part of the routine clinical management of patients in the health care facilities following the national guidelines.

## Results

### Baseline differences in serum IgE level, CD4^+^ and CD8^+^ T cell counts

The distribution of intestinal parasites detected and the demographic characteristics and immunological indices of the study groups are summarized in Tables 1 and 2. The mean age and male–female ratio in both groups was not different (Table 2). Although, it is very difficult to have a normal range of IgE at population level, a range of values of IgE levels was defined enabling the analysis of the frequency of normal, moderate and elevated IgE levels in each group of patients. Normal value (N) was adjusted and defined by the mean IgE levels from HIV negative apparently healthy helminths negative controls from the same geographic locale (562.5 IU/ml). Accordingly, serum IgE values between N and 1.5 N (562.5–843.75 IU/ml) were considered as low IgE levels and those between 1.5 N and 2 N (843.75–1,125 IU/ml) as moderate IgE levels, with the highest levels being above 2 N (>1,125 IU/ml).

**Table 1:** Distribution of intestinal parasite among acute sero-converters and symptomatic HIV/AIDS patients

Controls	Symptomatic HIV infectio (N = 60)	Asymptomatic HIV infection (N = 45)	Healthy (N = 25)
Heliminths			
AL	31	16	8
TT	11	5	3
HW	6	4	2
SS	3	1	0
SM	1	1	0
TS	3	1	1
HN	1	0	0
Protozoans			
GL	2	0	0
EH/D	3	0	0

AL, *Ascaris lumbricoides*; TT, *Trichuris trichiura*; HW, *Hookworm*; SS, *Strongyloide stercoralis*; SM, *Schistosoma mansoni*; TS, *Taenia saginata*; HN, *Hymenolopis nana*; GL, *Gardia lamblia*; EH/D, *Entaeamba histolytica/dispar*.

**Table 2:** Summary of demographic characteristics and immunological indices

Characteristics	Symptomatic HIV infection (N = 60)	Asymptomatic HIV infection (N = 45)	Healthy controls (N = 25)
Males	28	19	17
Females	32	26	8
Mean age (years)	30.6 ± 8	29.0 ± 9	26.5 ± 7
Median (IQR) total serum IgE (IU/ml)			
With helminths	1,593 (1,132–2,497)^b^	1,231 (706–2,241)^c^	908 (401–1,625)
Without helminths	1,259 (625–2,256)	831 (628–1,617)	662 (127–875)
Median (IQR) CD4^+^ T cells count (cells/mm^3^ of blood)			
With helminths	169 (48–201)	555 (128–672)^a^	707 (98–1,003)
Without helminths	161 (39–218)	677 (190–879)	792 (91–1,129)
Median (IQR) CD8^+^ T cells count (cells/mm^3^ of blood)			
With helminths	1,070 ± 103^d^	1,009 ± 82	1,072 ± 96
Without helminths	869 ± 87	813 ± 71	893 ± 84

^a^Significant difference compared to asymptomatic group without helminths and symptomatic group with helminths, *P* < 0.05.

^b^Significant difference compared to asymptomatic group with helminths and symptomatic group without helminths, *P* < 0.01.

^c^Significant difference compared with asymptomatic group without helminths, *P* < 0.01.

^d^Significant difference compared to symptomatic and asymptomatic group without helminths, *P* < 0.01.

**Figure 2:**
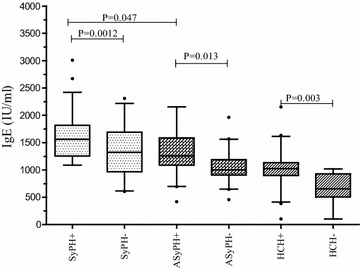
Serum IgE levels in adult HIV patients before initiation of ART and albendazole. *Horizontal lines* are medians and IQRs (25 and 75 centile). *Horizontal lines* are medians and interquartile ranges (IQRs) (25 and 75 centile) and the *black dot* represents outlier values. Keys: *SyPH±* Symptomatic HIV-1 infected patients with and without helminths infection, *ASyPH±* Asymptomatic HIV-1infected individuals with and without helminths infections, *HCH±* Healthy controls with and without helminths co-infection.

As indicated in Table 2 and Figure 2, irrespective of helminths infection individuals infected with HIV showed significantly high serum IgE level compared to HIV sero-negative apparently healthy controls (1,439, [IQR 498–2,771] versus 764 [IQR 134–1,683] IU/ml; P = 0.001). Similarly, HIV patients co-infected with helminths showed a high level of serum IgE compared to HIV patients without helminths co-infection (1,688 [IQR 721–2,473] versus 1,221 [IQR 618–2,289] IU/ml; P = 0.022). This difference was also markedly observed between symptomatic HIV infected patients with and without helminths infection (1,690 [IQR 1,116–2,491] versus 1,252 [703–2,251 IU/ml; P = 0.047). There was no statistically significant difference on the level of serum IgE level in HIV positive individuals co-infected with type of helminths (P = 0.07). However, individuals co-infected with multiple helminths had a higher mean serum IgE level than that of those infected with single helminth (1,994 ± 456 versus 1,554 ± 551 IU/ml). Among those infected with helminths irrespective of HIV infection, a statistically significant association was found between helminths egg intensity (representing “helminth egg load”) in stool and their serum IgE level. The mean baseline serum IgE level was significantly different between individuals with high or moderate (n = 12) helminth intensity and in individuals with low (n = 28) helminth intensity (2,156 versus 1,797 IU/ml; P = 0.025). In all groups the levels of serum IgE were not correlated with age and gender differences.

At baseline there was no significant difference in CD4^+^ T cell counts between symptomatic HIV-1 infected patients with and without helminth co-infection (169 [IQR 49–207] versus 166 ± 59 cells/mm^3^, respectively). However, the mean CD4^+^ T cell count in asymptomatic HIV-1 positive individuals with helminths was lower than that of those without helminth co-infection (577 ± 128 versus 635 ± 90 cells/mm^3^). Moreover, the mean level of CD8^+^ T cells count was significantly higher in those with helminth co-infected than those without helminth coinfected in both symptomatic HIV infected patient (1,070 ± 103 versus 869 ± 87 cells/mm^3^, P = 0.029) and asymptomatic HIV positive individuals (1,009 ± 82 versus 813 ± 71 cells/mm^3^, P = 0.014). A strong relationship is observed between IgE and the immune status as assessed by CD4 cell count of less than 200 or greater than 200 cells/mm^3^ i.e. 1,736 ± 231 versus 1,583 ± 171 IU/ml, P = 0.013.

### Impact of albendazole on serum IgE level, CD4^+^ and CD8 ^+^ T cells count

**Figure 3:**
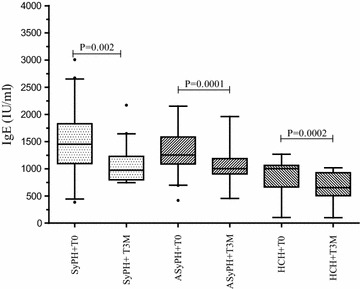
Comparison of changes in median total serum IgE at baseline (before treatment) and at 12 weeks after successful deworming in symptomatic, asymptomatic and health controls. *Horizontal lines* are medians and interquartile ranges (IQRs) (25 and 75 centile) and the *black dot* represents outlier values. Keys: Similar to Figure 2

As indicated in Figure 3, a significant decline in serum IgE level was observed 12 weeks after deworming and ART of symptomatic HIV infected patients with (1,487 versus 992, P = 0.002) and without (1,233 versus 976 IU/ml, P = 0.093) helminths co-infection. Similarly, serum IgE level was significantly reduced among asymptomatic HIV infected individuals (1,261 versus 996 IU/ml, P = 0.0001) and apparently health controls (993 versus 646 IU/ml, P = 0.0002) with helminths coinfection 12 weeks after deworming. However, there was no significant decrease in serum IgE level among asymptomatic HIV infected individuals (1,183 versus 1,097 IU/ml, P = 0.13) and apparently health controls (666 IU/ml versus 571, P = 0.09) without helminths co-infection 12 weeks after deworming. After controlling ART status and helminths coinfection a significant reduction in serum IgE level was observed in all groups (data not shown).

Patients with lower CD4^+^ T cell counts (<200 cells/mm^3^) showed a relatively lower reduction in the total serum IgE as compared with patients with higher CD4^+^ T cells count (>200 cells/mm^3^) after 12 weeks of either ART or albendazole (1,436–1,231 versus 1,583–1,107 IU/ml). Twelve weeks after albendazole treatment, a significant reduction in CD8^+^ T cells and an increase in CD4^+^ T cells count were observed (P = 0.0012). There was a significant reduction in CD8^+^ T cells count in symptomatic helminths co-infected patients 12 weeks after albendazole treatment (1,070 ± versus 872, P = 0.039). On the contrary, there was an a relative higher increase in mean CD4^+^ T cell count in patients with ART and albendazole treatment (from 168 to 249) as compared with patients with only ART (from 166 to 234) though the difference was insignificant. The mean serum IgE level among asymptomatic HIV positive individuals was increased in 12 weeks’ time from 1,321 ± to 1,544 IU/ml in the absence of both ART and albendazole and was parallel with the decrease in CD4^+^ T cells count from 677 to 596 cells/mm^3^.

## Discussion

In this study altered immune response as measured by total serum IgE level among symptomatic HIV infected patients, asymptomatic HIV positive individuals and healthy controls with and without helminths co-infection and the impact of deworming and/or ART on these immune activation was assessed within defined groups of population from tropical settings of Ethiopia where both HIV and helminths infections are common. Consistent with the earlier suggestions [25–27] that Africans generally present with elevated total serum IgE levels and our previous observations [14–16], patients in this study also showed a high total serum IgE level as shown by more than three-folds of the total IgE above the reference ranges irrespective of HIV and helminths co-infections. The highly significant correlation between helminth egg intensity and serum IgE level may partly explain the elevated total serum IgE levels observed in sub-Saharan regions, where heavy helminthic infections are widespread [14–16, 25–27]. It could be also associated with race-associated genetic mechanism that contributes for higher level of IgE in Blacks than Caucasians [28] and possibly due to the higher rate of malnutrition in Ethiopia [29] which in parallel increase in serum IgE.

We have previously shown that helminths infections cause immune activation by increasing memory CD4^+^ T cells which are reported to largely express CCR5 by the non-syncytium inducing HIV-1C subtype in Ethiopia [9]. The highly elevated serum IgE level in HIV patients with helminths co-infection in the current study might be a result of HIV-helminths induced immune dysregulation which provoke a shift in cytokine production from Th1 to Th2 [5, 30] and the resulting polyclonal activation of B cells which increase secretion of IgE [31, 32]. Although there may be other potential factors that could contribute to the increase in serum IgE level, it is known that IgE class switching is mediated by CD4^+^ Th2 cells [12, 13] and it is unclear whether the high IgE in African population is due to a genetic predisposition or environmental influences to mediate Th2 cell predominance [33]. Nevertheless, the altered of immune response during helimninths co-infection has been reversed to normal after deworming [this study, 6–8] among Ethiopians suggests that this decrease was indeed the result of helminthic infection alone without the influence of other environmental factors. Thus, the role of helminths in the host and the other infections cannot be overemphasized and should therefore be adequately addressed in managing HIV-infection during helminth coinfection [18, 21, 22, 34]. This is due to the fact that, helminths infections are potent stimulators of IL-4 dependent synthesis of helminths specific IgE [30].

The increased serum IgE level among symptomatic patients irrespective of helminths co-infections and the high level of IgE reduction 12 weeks after ART and deworming when compared with that of asymptomatic HIV infected individuals suggest that a Th2 response has occurred in advanced HIV/AIDS disease conditions [27]. Moreover, the increased serum IgE level among asymptomatic HIV positive individuals in 12 weeks time in the absence of both ART and albendazole is parallel with the decrease in CD4^+^ T cells count and could be associated with rapid diseases progression [14, 27] with time following the natural course of the diseases. A significant decline in serum IgE level 12 weeks after only deworming in the present study is inline with the finding that elimination or reduction of/in helminths resulted in a significant improvement in T cell proliferation and supports the notion ‘deworming of helminth co-infected individuals for delaying HIV disease progression’ [9, 10, 14, 17–22, 34]. Surprisingly, a significant decline in serum IgE level 12 weeks after simultaneous administration of ART and albendazole has been observed. This supports the fact that administration of ART reduces indices of immune activation [12, 13] and shows the presence of synergism between both drugs classes. However, it is difficult to ascribe the reduced IgE to only ART and albendazole in view of the fact that symptomatic HIV infected patients were also on cotrimoxazole prophylaxis and as the anti-helminthic effect of cotrimoxazole cannot be excluded [35]. Although the CD4^+^ T cell counts and recovery rates are lower among Ethiopians than in people from Caucasians irrespective of the HIV infection [9, 36, 37], an increased CD4^+^ T cell count and reduced CD8^+^ T cell count in HIV infected individuals with both ART and albendazole as compared with those from patients with ART was observed. The result shows the synergistic effects of ART and albendazole in down regulation of the polarized Th2 immune response.

Several studies have shown the impact of helminths on health, their consequences for the physical and intellectual development particularly in children, and the long-run effects on individuals. There is also sufficient evidence for the benefit of deworming on achieving healthy physical growth, improving cognitive domains, and the long-run effects on individuals [38]. Similarly, the negative impact of helminths infection on infectious diseases particularly on malaria, tuberculosis and HIV and the role of deworming on improving the quality of patient life and reducing morbidity and mortality have been documented [38]. Nevertheless, there is no routine screening for helminths infection and/or regular or mass deworming in most sub-Saharan African countries including Ethiopia even for HIV/AIDS patients.

### Limitations

This study has several limitations. First the obvious small sample size. Second, the short period (3 months of antiretroviral treatment-ART) of longitudinal observation which is relatively short to infer the mutual effect of ART and deworming on level of serum IgE. And, third the definition of allergy, which relies on self-reported and physician diagnosed allergy following the modified version of International Study of Asthma and Allergies in Childhood module. Forth, lack of specific IgE measurement before and after deworming. And lastly, although we and others have previously reported the association between eosinophil level and parasitic infection and correlated with higher level of total serum IgE, in this study we did not measure eosinophil count and could be considered as limitation of the study.

## Conclusion

The remarkably elevated IgE response in HIV patients irrespective of helminths co-infection suggests that HIV is a potent driver of IgE production like helminths. The significant decline of serum IgE level 12 weeks after administration of albendazole with and without ART in symptomatic patients indicate a shift in immune response towards Th1 which is crucial for prognosis of HIV patients. These are additional supportive evidence that deworming positively impacts HIV/AIDS diseases progression. Thus, mass deworming should be integrated with ART program in helminths endemic areas of tropical countries.
